# Allostatic load: a framework to understand breast cancer outcomes in Black women

**DOI:** 10.1038/s41523-021-00309-6

**Published:** 2021-07-30

**Authors:** Samilia Obeng-Gyasi, Willi Tarver, Ruth C. Carlos, Barbara L. Andersen

**Affiliations:** 1 Division of Surgical Oncology, Department of Surgery, The Ohio State University, Columbus, OH USA; 2 Department of Internal Medicine, College of Medicine, The Ohio State University, Columbus, OH USA; 3 Comprehensive Cancer Center, University of Michigan, Ann Arbor, MI USA; 4 Department of Psychology, The Ohio State University, Columbus, OH USA

**Keywords:** Breast cancer, Outcomes research

## Introduction

**Fig. 1:**
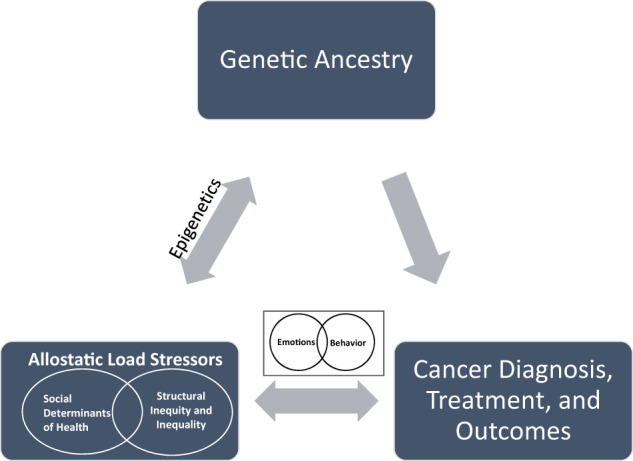
Allostatic load framework for understanding disparities in Black breast cancer patients. This figure describes the relationship between genetic ancestry, allostatic load stressors, and cancer diagnosis, treatment, and outcomes.

Examinations of patients across the cancer continuum show persistent racial disparities in diagnosis, access to care and mortality^1^. These disparities in presentation, treatment, and clinical outcomes (i.e., recurrence, mortality, treatment complications) are the result of interactions between social, behavioral, environmental, and biological exposures^2^. For Black breast cancer patients, these exposures are further contextualized within structural and systemic inequalities rooted in racism, sexism, and social class^3^. The objective of this commentary is to propose allostatic load as a framework to understand and measure disparities in breast cancer across the continuum from diagnosis through survivorship (Fig. 1). Moreover, Allostatic load provides a multidimensional framework that integrates the physiologic implications of structural inequity and inequality within the setting of biological, environmental, social, and behavioral factors^2^.

## Overview of racial disparities in breast cancer

Significant advances in breast cancer diagnosis, treatment, and survival have not translated into improvements in survival for Black women compared to their White counterparts^4^. Specifically, Black women are diagnosed with advanced stages of breast cancer, higher rates of aggressive subtypes, and are less likely to complete recommended systemic and locoregional therapies^4,5^. The causative agents of these disparities likely include an interplay between social determinants of health (SDH) rooted in structural inequity and systemic inequality, increased oncological risk profiles secondary to genetic ancestry and underlying tumor biology^5^. To date, the majority of research attempting to understand the persistent racial disparities in breast cancer clinical outcomes have used frameworks that focus on elements of socioeconomic factors, genetic ancestry, clinical variables (i.e., tumor stage, subtype), or treatment differences in isolation. Thus far, studies have neither integrated structural inequity or systemic inequality nor studied their effects prospectively to understand their role in clinical outcomes for breast cancer patients. This knowledge gap is significant as prior studies suggest living in a society with sociocultural norms resulting in sociopolitical and economic marginalization accelerates health deterioration^6^. The current approach to describing and defining racial disparities in breast cancer has resulted in an incomplete understanding of the contributions and relationships among structural inequity, systemic inequality, psychosocial, environmental and genetic factors on presentation, treatment, and clinical outcomes. Consequently, we propose allostatic load as a framework to define, measure, and understand the implications of structural inequity and systemic inequality on the racial disparities Black breast cancer patients experience across the cancer continuum.

## Stress, cancer, and allostatic load as a framework for structural inequity and systemic inequality

Psychosocial stressors have been implicated in tumor initiation and progression. The proposed pathway includes the release of primary stress hormones such as cortisol and catecholamines by the hypothalamic–pituitary–adrenal axis (HPA) and the autonomic nervous system (ANS) respectively^7^. Stress hormones influence the tumor microenvironment by decreasing the immune response, increasing cancer cell migration and invasion while concomitantly stimulating angiogenesis^7^.

In addition to tumorigenesis, stress hormones have effects on the cardiovascular (e.g., regulation of blood pressure and heart rate by catecholamines) and metabolic systems (e.g., increased gluconeogenesis due to elevated cortisol) that has implications for chronic illnesses such as cardiovascular disease and diabetes^7^. The non-tumorigenic physiologic effects of stress hormones, in the setting of acute stressors, are an adaptive response termed Allostasis^8^. Allostasis describes an appropriate physiologic response to a stressor and a return to baseline upon resolution of the stressor^8^. Conversely, persistent physiologic dysregulation secondary to chronic psychosocial stressors is called allostatic load (AL)^8^.

AL describes how the chronic activation of the stress response in the setting of elevated psychosocial stressors (e.g., neighborhood deprivation, social isolation, financial hardship, unemployment) leads to physiologic dysregulation and subsequent increased risk for illness such as cancer, obesity, diabetes, and heart disease^9^. AL provides a unique framework to understand and measure the implications of chronic stress as mediated through environmental and psychosocial factors on health status and health outcomes^9^. Furthermore, it enables the operationalization of the cumulative physiologic impact of deprivation driven by structural inequity and systematic inequality^6^. At its core, AL frames the multisystem biologic stress responses of the HPA and the autonomic nervous system on the development of disease and response to treatment^9^.

Currently, there are no specific biomarkers used in the calculation of AL. Instead, AL is operationalized with a composite score of measures of primary stress mediators (i.e., cortisol), secondary outcomes from the primary mediators (i.e., C-reactive protein, glycosylated hemoglobin) and downstream health outcomes (e.g., heart disease, diabetes, cancer)^9,10^. Biomarkers for AL include ones of the neuroendocrine (e.g., cortisol), cardiovascular (i.e., systolic blood pressure, diastolic blood pressure, triglycerides), metabolic (body mass index, creatinine, fasting blood glucose), and the immune system (white blood cell count, C-reactive protein)^9,11^. Higher AL scores are interpreted as reflecting greater physiologic dysregulation secondary to external stressors.

Studies evaluating AL suggest an association between external stressors such as poverty, financial hardship, increasing job demands and lower education attainment and elevated AL^9,12–14^. Moreover, there are age, racial, and sex-based differences in AL with older age, Black race, and female sex being associated with elevated AL^6,15,16^. For instance, in Nelson et al.’s examination of a multiethnic cohort, there was an association between high AL, Black race, and peripheral artery disease^17^. These study results are unsurprising as current United States sociocultural, political, and economic norms create social hierarchies that adversely affect health outcomes in the aforementioned groups^18,19^. Additionally, elevated AL has been implicated in physical deterioration and cognitive decline in the elderly and an increased all cause and disease specific mortality among cancer patients^8,20^. Taken together, these findings suggest AL could provide an avenue to evaluate, measure, and operationalize environmental, structural, and psychosocial sources of stress on clinical outcomes in populations facing structural inequity and systemic inequality^21^.

### Allostatic load and Black breast cancer patients

Multiple studies have evaluated the relationships between AL, socioeconomic factors, and other chronic illnesses (cardiovascular disease etc.), but there is a paucity of literature on AL among patients with cancer^9,22^. Moreover, as of the writing of this paper, only four studies have evaluated AL in Black breast cancer patients (Table 1). Two studies reported race-related differences: Black patients had higher AL than did white patients^16,23^. This finding is consistent with other non-cancer studies showing Black women having higher AL than their White female counterparts^6^. Studies have also reported an association between elevated AL, Black race, poor tumor prognostic features (large tumor size and poor tumor differentiation), and aggressive subtypes (estrogen receptor negative)^16,24^. In a repeated measures test of AL and patient reported outcomes, the Functional Assessment of Cancer Therapy-General (FACT-G) and Functional Assessment of Cancer Therapy-Breast Cancer (FACT-B), high AL was associated with lower FACT-G scores and the functional well-being subscale in the FACT-B^25^. Collectively, these are important “early data”, suggestive of interactions among structural inequity, systematic inequalities, AL, and clinical outcomes among Black breast cancer patients.

**Table 1:** Summary of studies including Black breast cancer patients and allostatic load (AL) biomarkers.

Refs.	*N*	Study design and subjects	AL biomarkers with cut-offs for AL determination	Composite AL score cut-offs	Key findings
Parente et al.^23^	4875	Cross-sectional; Black and White women, ages 35–85	1. Systolic blood pressure ≥140 mmHg 2. Diastolic blood pressure ≥90 mmHg 3. Heart rate ≥90 beats; per minute 4. Total cholesterol level ≥240 mg/dL 5. High-density lipoprotein cholesterol <50 mg/dL 6. Body mass index ≥30 kg/m^2^ 7. Glycosylated hemoglobin ≥6.4% 8. C-reactive protein >3 mg/L 9. Albumin <4 g/dL.	Low Al ≤3, High AL ≥4	A history of breast cancer was associated with elevated AL among Black women but not their white counterparts, adjusting for age, income, education, insurance type, and smoking history.
Xing et al.^24,25^^a^	AL Lipid 229 AL Inflammatory 409	Cross-sectional; Black women, ages 20–75	*Lipid profile:* 1. Systolic blood pressure ≥140 mmHg; 2. Diastolic blood pressure ≥90 mmHg 3. Waist circumference ≥88 cm 4. Glucose level ≥110 mg/dL 5. HDL <50 mg/dL 6. Total cholesterol >240 mg/dL or total cholesterol ≤240 mg/dL and LDL >130 mg/dL 7. Triglycerides ≥150 mg/dL 8. Ever use of medications to control hypertension, diabetes, or hypercholesterolemia *Inflammatory profile*: 1. Systolic blood pressure ≥140 mmHg; 2. Diastolic blood pressure ≥90 mmHg 3. Waist circumference ≥88 cm 4. Glucose level ≥110 mg/dL 5. HDL <50 mg/dL 6. Total cholesterol >240 mg/dL or total cholesterol ≤240 mg/dL and LDL >130 mg/dL 7. Triglycerides ≥150 mg/dL 8. Ever use of medications to control hypertension, diabetes, or hypercholesterolemia 9. eGFR <59 mL/min 10. Albumin <4 g/dL 11. BMI _30 kg/m^2^	Low Al ≤3, High AL ≥4	High pre-diagnostic AL, calculated by the lipid and inflammatory profiles, was associated with higher tumor grade. Elevated AL, calculated by the inflammatory profile, was associated with a larger tumor size. Elevated pre-diagnostic AL, calculated by inflammatory profile, associated with lower scores on the Functional Assessment of Cancer Therapy-General (FACT-G) survey and the functional well-being subscale assessed by the Functional Assessment of Cancer Therapy-Breast Cancer (FACT-B) survey.
Zhao et al.^16^	934	Cross-sectional: Black and White women, aged 20–60+	1. SBP ≥140 mmHg 2. DBP ≥90 mmHg 3. HDL <50 mg/dL 4. Total cholesterol >240 mg/dL or total cholesterol ≥240 mg/dL 5. LDL >130 mg/dL 6. Triglycerides ≥150 mg/dL 7. Waist circumference ≥88 cm 8. BMI ≥30 kg/m^2^ 9. Glucose level ≥110 mg/dL 10. HbA1C >6.5 11. Albumin <4 g/dL 12. CRP >3 mg/L 13. IL-6 >1.8 pg/mL 14. eGFR <60 mL/min/1.73m^2^; (14) 15. Creatinine >1.2 mg/dL 16. RHR >100 bpm 17. Previously taking medications to control metabolic diseases and hypertension	Low AL, 0–8 High AL, 9–16	AL was higher in Black and Hispanic patients compared to White patients AL was high in Black patients after adjusting for age at diagnosis, marital status, education, smoking status, alcohol status, physical activity, and tumor stage. There was an association between high AL and poorly differentiated tumors across all study races and ethnicities. In Black patients, there was an association between high AL and estrogen receptor negative tumors.

^a^This summarizes two articles by Xing et al.

Unfortunately, the rigor of these studies is limited by multiple factors. There is heterogeneity in the calculation of AL making it difficult to compare results across data sources. Moreover, none of the calculations of AL used have been validated in breast cancer patients nor have they been replicated in other studies. Since these data come from secondary analyses with large data sets, the biomarkers used for AL were limited by variables originally included. The data are correlational, preventing causal inferences between stressors, AL, and outcomes. To this end, the implications of these findings on clinical outcomes and the integration of AL into clinical practice requires further investigation. Nevertheless, study results suggest AL may influence breast cancer outcomes which warrants additional inquiry.

## Addressing research gaps in AL and breast cancer

To address the gaps in AL research in breast cancer, key biomarkers need to be established and validated to measure AL. Such would enable comparison across studies and accumulation of data to evaluate the reliability of AL effects. Beyond correlation analysis, future prospective studies should focus on understanding if AL functions as a mediator or moderator of the effect of structural inequity and systemic inequalities on clinical outcomes. Currently, AL is believed to function through a bifactor model^26^. This suggests AL represents a common factor for its constituents in conjunction with the individual constituents of AL acting independently of AL. Defining AL’s role as a moderator or mediator will help explain the relationship between biomarkers and the strength and direction of those relationships individually and as a composite score.

There are few AL studies with longitudinal designs. Consequently, studies with longitudinal designs are needed to enable measurements of AL at multiple time points. Clinical trials may offer an important venue to explore the relationships between AL and clinical outcomes as following accrual, treatment(s) type is controlled and evaluated. Additionally, there are established mechanisms for the collection of blood samples as correlatives and patient reported outcomes (Box 1).

Box 1: Recommendations to enable integration of allostatic load into clinical trials

1. Use AL biomarkers standardized according to disease site and underlying pathophysiology.
2. In all oncology clinic trials, collect AL biomarkers to enable calculation of AL and test relationships to study endpoints (survival, recurrence, tolerability, and trial completion).
3. Collect self-reported race, ancestry, and social determinants of health (e.g., employment, financial hardship, marital status etc.) in conjunction with AL in all clinical trials.

## Conclusions

Racial differences in breast oncologic outcomes persist and show little abatement^4^. Despite extensive research on racial differences in clinical outcomes (e.g., mortality), longitudinal studies have not been done to study the biologic impact of structural inequity and systemic inequality on breast cancer outcomes. Early studies on AL in Black breast cancer patients are suggestive of physiologic dysregulation secondary to external and systemic stressors playing a role in breast cancer outcomes. The routine collection of AL in the delivery of oncology care would be transformative for a comprehensive understanding of the intersectionality of race and gender that Black women face and its interplay with structural inequity, systemic inequality, and clinical outcomes.

## Data Availability

Data sharing not applicable to this article as no datasets were generated or analyzed during the current study.

## Code availability

code availability not applicable to this article as no custom code or mathematical algorithms were generated or analyzed during the current study.
